# Complexity of Chemical Emissions Increases Concurrently with Sexual Maturity in *Heliconius* Butterflies

**DOI:** 10.1007/s10886-024-01484-z

**Published:** 2024-03-13

**Authors:** Bruna Cama, Karl Heaton, Jane Thomas-Oates, Stefan Schulz, Kanchon K. Dasmahapatra

**Affiliations:** 1 Department of Biology, University of York, Wentworth Way, Heslington, YO10 5DD UK; 2 Department of Chemistry, University of York, Heslington, YO10 5DD UK; 3 Institute of Organic Chemistry, Technische Universität Braunschweig, Hagenring 30, Braunschweig, 38106 Germany

**Keywords:** Pheromones, *Heliconius*, Aging, GC-MS, Androconia, Antiaphrodisiac, Lepidoptera

## Abstract

Pheromone communication is widespread among animals. Since it is often involved in mate choice, pheromone production is often tightly controlled. Although male sex pheromones (MSPs) and anti-aphrodisiacs have been studied in some *Heliconius* butterfly species, little is known about the factors affecting their production and release in these long-lived butterflies. Here, we investigate the effect of post-eclosion age on chemical blends from pheromone-emitting tissues in *Heliconius atthis* and *Heliconius charithonia*, exhibiting respectively free-mating and pupal-mating strategies that are hypothesised to differently affect the timing of their pheromone emissions. We focus on two different tissues: the wing androconia, responsible for MSPs used in courtship, and the genital tip, the production site for anti-aphrodisiac pheromones that affect post-mating behaviour. Gas chromatography-mass spectrometric analysis of tissue extracts from virgin males and females of both species from day 0 to 8 post-eclosion demonstrates the following. Some ubiquitous fatty acid precursors are already detectable at day 0. The complexity of the chemical blends increases with age regardless of tissue or sex. No obvious difference in the time course of blend production was evident between the two species, but female tissues in *H. charithonia* were more affected by age than in *H. atthis*. We suggest that compounds unique to male androconia and genitals and whose amount increases with age are potential candidates for future investigation into their roles as pheromones. While this analysis revealed some of the complexity in *Heliconius* chemical ecology, the effects of other factors, such as the time of day, remain unknown.

**Supplementary Information:**

The online version contains supplementary material available at 10.1007/s10886-024-01484-z.

## Introduction

All living organisms utilize signals to communicate with and elicit responses from other individuals. In animals, these signals may convey different messages (e.g., readiness to mate, aggression, aggregation) and come in a number of different forms depending on which aspect of the recipient’s perception they target (e.g., visual, acoustic, olfactory), while the receiver may be conspecific or heterospecific to the signaller (Laidre and Johnstone 2013). Regardless of their function, most signals are heavily context dependent, as the physiological state of the signaller, that of the receiver, and even the external environment may affect how information is conveyed and how it is perceived (Endler 1992; Laidre and Johnstone 2013). For this reason, the timing of the signals is important in order for information to be appropriately interpreted, and most species do not signal continuously but rather transmit under specific conditions and at specific moments in their life cycle (Endler 1992).

Chemical signals are ubiquitous across the tree of life (Hardege et al. 1998; Snell 1998; Tillman et al. 1999; Gosling and Roberts 2001; Casselton 2002; Stacey 2003; Jacob et al. 2004; Grammer et al. 2005; Sekimoto 2005; Luporini et al. 2015; Dong et al. 2020), and among them we find pheromones (Wyatt 2009). Defined as blends of compounds that mediate communication between conspecific individuals by affecting the recipient’s behaviour (Karlson and Lüscher 1959), pheromones are among the most important signals in the animal kingdom, mediating several decisions and behaviours adopted by the organism during its life cycle (Yew and Chung 2015). Pheromones can be used as a means to differentiate conspecific from heterospecific individuals, leading to their composition being species-specific in these cases (Yew and Chung 2015), or even race-specific in some known instances (Kochansky et al. 1975; Glover et al. 1987; Bengtsson et al. 2014). This specificity is not only derived from the identity of the blend components, but also their ratios, with receiving individuals being able to perceive differences in composition and titre (Klun et al. 1973; Glover et al. 1987). On top of often communicating the species identity of the signalling individual to all the receivers, pheromones may have a function in courtship, mate choice, aggregation, aggression and even social structure (Johansson and Jones, 2007; Wyatt 2009; Yew and Chung 2015). Thus, the context in which pheromones are emitted and the physical conditions of the signalling individual are extremely important. Insects have been shown to be able to discern a conspecific’s age, mating status and sex based on their chemical signatures (Cuvillier-Hot et al. 2001; Everaerts et al. 2010; Nieberding et al. 2012; Heuskin et al. 2014; Polidori et al. 2017), and the timing and composition of pheromone emissions themselves are regulated by factors such as age and circadian rhythms, as well as feeding regime (Raina et al. 1986; Rafaeli and Soroker 1989; Larsdotter-Mellström et al. 2012; Nieberding et al. 2012; Heuskin et al. 2014; Ruther et al. 2014; Darragh, Byers, et al., 2019; Pokorny et al. 2020). This means the chemical signals potentially carry large amounts of information that the conspecifics are able to decode and interpret (Johansson and Jones, 2007).

In Lepidoptera (moths and butterflies), pheromones are widely used during courtship and mating (Roelofs and Rooney 2003; Yew and Chung 2015). Female moths often emit long distance sex pheromones that attract mate-seeking males (Zhang and Löfstedt 2015; Allison and Carde 2017), whereas in butterflies, males use volatile male sex pheromones (MSPs) during courtship (Myers 1972; Nieberding et al. 2008). The receiving individuals perceive these signals, and likely use them to assess the prospective mate’s quality, a process that is still poorly understood (Johansson and Jones, 2007) and may take into account such parameters as species identity, size, or general health. In turn, this assessment influences their decision to mate (Andersson et al. 2007; Johansson and Jones, 2007; Darragh et al. 2017). These signals are not the only type of Lepidopteran chemical cue to affect mating behaviour, as some species also produce anti-aphrodisiacs. These are physically delivered from males to females through their genitals during mating, and signal the mated status of the female, thereby reducing competition and harassment from other males (Andersson et al. 2000, 2003; Schulz et al. 2008; Estrada et al. 2011). Female butterflies can utilize the males’ pheromone composition to discern their age (Nieberding et al. 2012; Karl et al. 2013), and this information is known to affect mate choice in at least some species, not only in butterflies, but in other animals as well (Drickamer and Brown 1998; López et al. 2003; Coppée et al. 2011; Nieberding et al. 2012; Karl et al. 2013).

*Heliconius* butterflies are model organisms in evolutionary biology (Merrill et al. 2015), and interest in research on their chemical signals has only begun growing in the past decade, with studies on both their MSPs, emitted during courtship from specialized scales on the male wings known as androconia, which are absent in females (Vanjari et al. 2015; Darragh et al. 2017, 2020; Mann et al. 2017; Cama et al. 2022), and their genital anti-aphrodisiacs (Gilbert 1976; Schulz et al. 2008; Estrada et al. 2011; Darragh, Orteu, et al., 2019; Byers et al. 2020). In *Heliconius*, the chemical blends produced in these tissues include host plant-derived metabolites such as terpenes and aromatics, as well as endogenously produced terpenoids and fatty acid (FA) derivatives (Mann et al. 2017; Cama et al. 2022), derived mainly from three fatty-acid precursors (palmitic, stearic and oleic acid) via a metabolic pathway that is conserved among Lepidoptera (Tillman et al. 1999; Liénard et al. 2014; Yew and Chung 2015). Female *Heliconius* are known to participate actively in mate choice; while they can use pheromone blends to recognize members of their own species (Darragh et al. 2017; Southcott and Kronforst 2018; González-Rojas et al. 2020), they also choose to reject or accept copulation from conspecific males, a decision thought to be mediated by MSPs through as-yet poorly explored mechanisms. Understanding the effect of endogenous (e.g. age, mating status, adult and larval food sources) and exogenous (e.g. time of the day, temperature, presence/absence of conspecifics or competitors) factors on male *Heliconius* pheromone composition may prove crucial to understanding how females interpret these signals.

*Heliconius* butterflies are typically long lived (Brown 1981), with a lifespan of over 6 months, but compared to their long life cycle they are known to reach reproductive age relatively early: males become sexually active ~1 week post-eclosion (Mérot et al. 2015; Rosser et al. 2019) while females are able to mate immediately upon eclosion. While these are common features across all *Heliconius* species, the genus can be divided into two monophyletic lineages that are known to show two different mating strategies. Some species are “free-mating” and commence copulation after male courtship, with females potentially mating multiple times over their lifetimes. But in species belonging to the “pupal-mating” clade (Gilbert 1976; Deinert et al. 1994; Beltrán et al. 2007), males compete amongst themselves to guard female pupae (which usually hang from the lower surface of leaves), mating with females soon after or even during eclosion (Deinert et al. 1994), which they achieve by inserting their abdomen within the female pupa as the pupal cuticle breaks open during eclosion. This behaviour, while frequently observed, is not obligate and chiefly depends on the availability and proximity of pupae (Klein and de Araújo 2010). While the pupae themselves emit chemical signals to attract males (Estrada et al. 2010), females mated as pupae cannot actively partake in mate choice, and once copulation is complete they are very unlikely to re-mate (Walters et al. 2012). This leads to a few key differences in reproductive behaviour, with free-mating females believed to be promiscuous and pupal-mating females believed to be monoandrous (Walters et al. 2012). These differences in mating strategy might affect the timing of pheromone production. The reduced post-mating competition (Estrada et al. 2011) may mean that males from pupal-mating species invest less in anti-aphrodisiacs than males from free-mating species, with their volatile genital contents showing no correlation with the onset of sexual maturity and fewer differences in composition between sexes. Conversely, freshly emerged female tissues might be expected to contain male attracting volatiles related to the pupal-mating behaviour, potentially similar to those described in (Estrada et al. 2010). Similarly, since no courtship is involved prior to mating, males from pupal-mating species may invest less in MSP production compared to free-mating males, and therefore their androconia might contain fewer unique and age-dependent compounds.

As age has been shown to commonly affect the titre, composition and/or perception of volatile and cuticular chemical signals in arthropods (Raina et al. 1986; Tregenza et al. 2000; Cuvillier-Hot et al. 2001; Schulz et al. 2008, 2013; Estrada et al. 2010; Cory and Schneider 2016; Domínguez et al. 2019; Pokorny et al. 2020), knowing the effect of this parameter on *Heliconius* chemical emissions is important and has the potential for being useful for future experimental designs centred around courtship behaviours and physiological changes associated with sexual maturity and mating. A past study on *H. melpomene* revealed a stark difference in volatile composition in the genitals of males at two different time points (freshly eclosed and 5 days post-eclosion) (Schulz et al. 2008), a finding which highlights the importance of determining a clear timeline of volatile production. Importantly, this information may provide insight into how females discriminate between different conspecific males when it comes to mating. Furthermore, exploring the amounts of different compounds may provide insight into how pheromone biosynthesis is carried out based on whether or not the precursors are accumulated within the same tissues as the products as is the case for other Lepidopteran species (Liénard et al. 2014).

Here, we investigate the effect of age on the volatile chemical contents of two male *Heliconius* pheromone-producing tissues: the androconia responsible for MSPs and the genitals, which produce antiaphrodisiac pheromones, alongside analysis of female tissue samples from equivalent regions. These tissues are investigated in the free-mating species *Heliconius atthis*, and the pupal-mating species *Heliconius charithonia* (Estrada et al. 2010; Walters et al. 2012) to answer the following questions: (1) Is age a good predictor for the composition of androconial chemical blends, and how does it affect the number and type of compounds produced? (2) How many and which compounds are age-dependent and does this vary with sex? (3) Do precursor compounds appear before the final components of the mature blend? (4) Is there any difference in the effect of age between the two different *Heliconius* mating strategies, given their different mating dynamics?

## Methods

### Specimen and Tissue Extraction

All specimens used in this study were purchased from Stratford Butterfly Farm (Stratford-upon-Avon, United Kingdom) as pupae, sourced from populations in Ecuador and Costa Rica. The chosen species were *Heliconius atthis* for the free-mating clade and *Heliconius charithonia* for the pupal-mating clade. On eclosion, adults had their wings marked to allow individual identification. Adult butterflies were housed in large insectaries at the University of York (UK) greenhouses under natural lighting from early June to late August 2019 at a temperature of at least 25 °C, and fed on a mixture of water, honey and pollen. The two species were housed separately in a set of 1.5 m (W) × 1.5 m (L) × 2 m (H) mixed sex cages to allow for natural interactions between males and females, and kept under observation during daylight hours.

The sampling protocol was the same for individuals from the two species and sexes. Based on courtship and mating activity, male *Heliconius* are known anecdotally to reach sexual maturity around ~ 7–8 days post-eclosion (Mérot et al. 2015; Rosser et al. 2019; Rosser & Mallet 2021, Personal Communication, 20 Dec.), so individuals were sacrificed at five different ages: 0, 2, 4, 6 and 8 days after eclosion. For each time point we sampled between 2 and 5 individuals of each sex, and from each individual we dissected samples of three different tissues: (1) the wing androconia in males, responsible for emitting the courtship pheromones (Darragh et al. 2017; Rosser et al. 2019) and equivalent regions from the females, referred to as female “androconia” (this wording is used purely to highlight correspondence between the male and female tissues, as these regions do not actually contain any known pheromone-emitting structures in females); (2) a non-androconial control region of the wing, of comparable area to the androconia; (3) the last abdominal segment: in males, this is where claspers are located, which carry the antiaphrodisiac producing glands, whereas females possess complementary structures to the claspers wherein these scent bouquets are deposited (Eltringham 1925; Schulz et al. 2008; Estrada et al. 2011). The samples were dissected using sterile forceps and scissors and immediately suspended in 200 µL dichloromethane containing 1 ng/µL 2-acetoxytetradecane as the internal standard (IS), in a 2 mL glass vial, where they were left to soak for 12 h. The known concentration of the IS was used to calculate the relative amount of other compounds (Supp. Material 2) as described in (Ehlers et al. 2023), based on peak intensities. These relative intensities are proportional to the compound mass and an accurate proxy for it.

Two of the day-6 *H. charithonia* females had mated, but were included in the study as their genital contents showed no difference in composition to those of unmated females.

### Gas Chromatography-Mass Spectrometry (GC-MS) Settings and data Processing

Tissue extracts from both sexes were analysed on a GC-MS system consisting of a 7890 A GC-System (Agilent Technologies, Santa Clara, CA, USA) coupled with a Waters GCT Premier TOF Mass Analyzer (Waters Corporation, Milford, MA, USA) fitted with a Phenomenex ZB5-MSplus (30 m x 0.25 mm x 0.25 μm) column (Phenomenex, Macclesfield, UK). Note that with this column type, fatty acids tend to form irregularly shaped GC peaks due to tailing, an issue which was addressed by the deconvolution software PyMassSpec, known for its accuracy in the presence of complex GC peaks (O’Callaghan et al. 2012). To improve mass accuracy, MS grade perfluorotributylamine (Heptacosa, Code: PC0568; Apollo Scientific Ltd., Stockport, UK) was utilized as a constant-flow calibrant. Electron ionisation with an electron energy of 70 eV was used. The instrument settings were the following: inlet pressure 67.5 kPa, inlet type split/splitless, transfer line temperature 280 °C, split ratio 20:1, carrier gas He 20 mL min^− 1^, injection volume 1 µL. The GC was programmed with the following temperature ramp: starting at 50 °C, increased by 5 °C min^− 1^ to a maximum temperature of 320 °C, then held for 5 min for a programme total of 69 min. A C6-C40 alkane calibration mixture was used to calculate the linear retention index (RI) for each compound.

### Data Processing

The GC-MS data files were converted into CDF format and processed via untargeted analysis using the Python package PyMassSpec (Davis-Foster 2019, originally from O’Callaghan et al. 2012). A large number of chemical species found in *Heliconius* had been previously described in multiple studies (Estrada et al. 2011; Darragh et al. 2017; Mann et al. 2017; Ehlers et al. 2021; Cama et al. 2022) conducted at TU Braunschweig (Braunschweig, Germany) and compiled in libraries of mass spectra, so compound identification was carried out in AMDIS (Stein 1999) by comparing mass spectra of the chemical species found in this study with those reported in said libraries. For further confirmation, retention indices were also cross-referenced with those reported in existing databases, particularly the NIST Mass Spectra and Retention Indices databases (Linstrom and Mallard 2021), for the same or suitably similar column type as the ZB5-MSplus utilized in our instrument. In case of compounds previously unreported in *Heliconius*, identification was also carried out at TU Braunschweig via direct analysis of mass spectra coupled with comparisons against a wider variety of insect libraries. All analyses were run in R using the base stats package. Genital extract data were analyzed separately from wing tissue (androconia and control) extract data. Thus, 6 datasets were generated for each species: male androconia, male control, male genitals, female “androconia”, female control and female genitals.

### Data Analysis

Simple linear models were used to test for changes in the number of detected compounds and the total amount of tissue contents (signal intensity) over time. This was carried out for each tissue type and for each species separately. Linear models were also used to test the effect of age on each individual compound’s amount separately for the six tissue types per species. The resulting p-values were corrected using the Benjamini-Hochberg procedure for controlling the false discovery rate (Benjamini and Hochberg 1995).

To test whether age is a good predictor of pheromone composition, the dimensionality of the data was first reduced using the metaMDS function in the vegan package (Dixon 2003) on each of the six tissue types per species, with k = 2 and based on 1000 permutations of the data. A linear discriminant analysis (LDA) model was then run on the two resulting NMDS axes, NMDS1 and NMDS2, to test the extent to which global pheromone composition was different between each age class in each of the six different tissue types per species (male androconia, male control, male genitals, female “androconia”, female control, female genitals). This model was trained using a partition of 60% of the dataset and its accuracy was calculated using the jackknife (leave-one-out) cross-validation approach.

To better discern potential pheromonal compounds and functionally relevant matrix components from structural components, we then performed an Indicator Compound Analysis (ICA) using the R(indicspecies) (De Cáceres and Legendre 2009) package for every tissue separately, using only 8 day-old individuals assuming that these carry the blend of compounds characteristic of sexually mature adults. Indicator compounds of male androconia that are not found in other wing tissues of either sex may be good candidates as male sex pheromone components, and indicator compounds of male genitals that are not found in female genitals may be good antiaphrodisiac candidates. The ICA tests for patterns of occurrence for individual compounds across the different tissues (Heuskin et al. 2014; Darragh et al. 2020), and for each compound calculates the tissue specificity and sample coverage (both values ranging from 0 to 1). An indicator value (IV) can be calculated from the square root of the product of specificity and coverage (De Cáceres and Legendre 2009). An IV of 1 for a compound within a tissue type indicates that the compound is found in all samples of that type (coverage = 1) and in no other tissue types (specificity = 1). Nonetheless an indicator compound does not necessarily require IV = 1, and lower values are admissible as long as the association between compound and sample type is significant, based on a permutation test. IV calculation and significance testing was carried out using the indicspecies function “multipatt”.

## Results

**Fig. 1:**
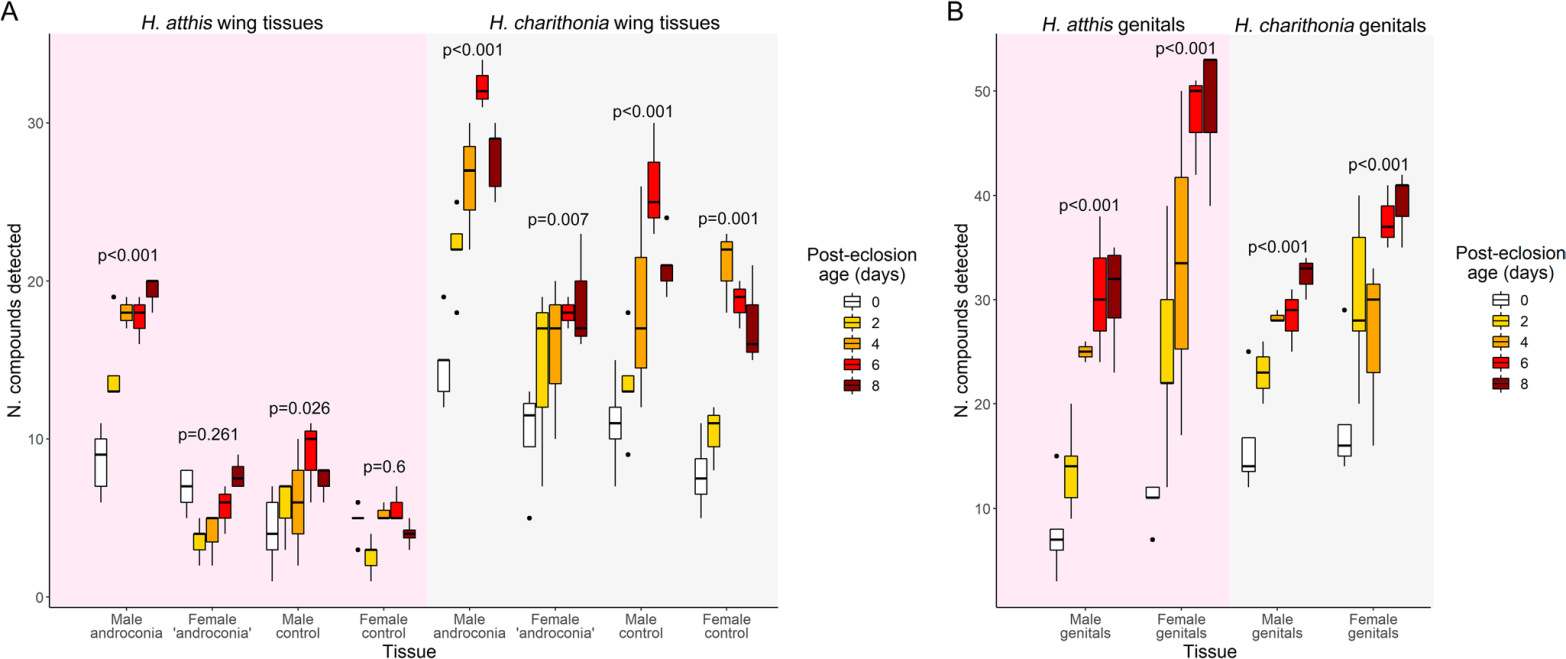
Effect of age on number of compounds detected in *H. atthis* and *H. charithonia* (**A**) wing tissue extracts and (**B**) genital extracts at all measured time points, also showing the data distribution for each time point in each tissue. Above each set are the linear regression *p-*values for the effect of age on number of compounds. See Table S2 for the complete linear regression results including all test statistics, as well as results for the effect of age on the total amount of all detected compounds, and Figure S2 for a plot of the effect of age on the total amount of all detected compounds

A total of 225 samples were analyzed by GC-MS, encompassing three tissue types (androconia, wing control and genitals) across 5 time points (0, 2, 4, 6, 8 days post-eclosion) for both species; 18 male and 20 female *H. atthis*, and 21 male and 16 female *H. charithonia*. Between three and five samples were collected for all tissues at each time point, the only exception being 6 days old *H. atthis*, for which only two individuals could be recovered. Compounds identified include fatty acids and FA-derivatives as well as terpenes and aromatic compounds, the latter two categories potentially comprising both plant-derived and endogenously produced (Agerbirk et al. 2010; Darragh, Orteu, et al., 2019) chemical species. A greater variety of compounds were detected in *H. charithonia* wing tissue extracts, regardless of sex, than in those of *H. atthis* (Fig. 1A), with male androconia containing on average more compounds than any other wing tissue. The number of compounds detected in the genital extracts was instead comparable between the two species. For a full list of compounds detected in all samples of the dataset, see Supp. Information 2.

### Global Effect of Age on Chemical Blend Composition

A linear model was run on the results of the analysis of extracts of each tissue from each species separately, to determine whether age affects the number of different compounds detected and the total amount, estimated from the summed mass of all detected compounds. We found that the number of different compounds detected in the tissues increases with age in both species: in *H. atthis*, it does so in all male tissues as well as female genitals, but not in female wing tissues, whereas in *H. charithonia* it shows a correlation with age in all three tissue types in both sexes (Fig. 1). The total amount of contents correlates positively with age only in male androconia of both species, in male *H. atthis* genital extracts and in *H. charithonia* female genitals, and correlates negatively with age in *H. atthis* female control (Table S2).

**Fig. 2:**
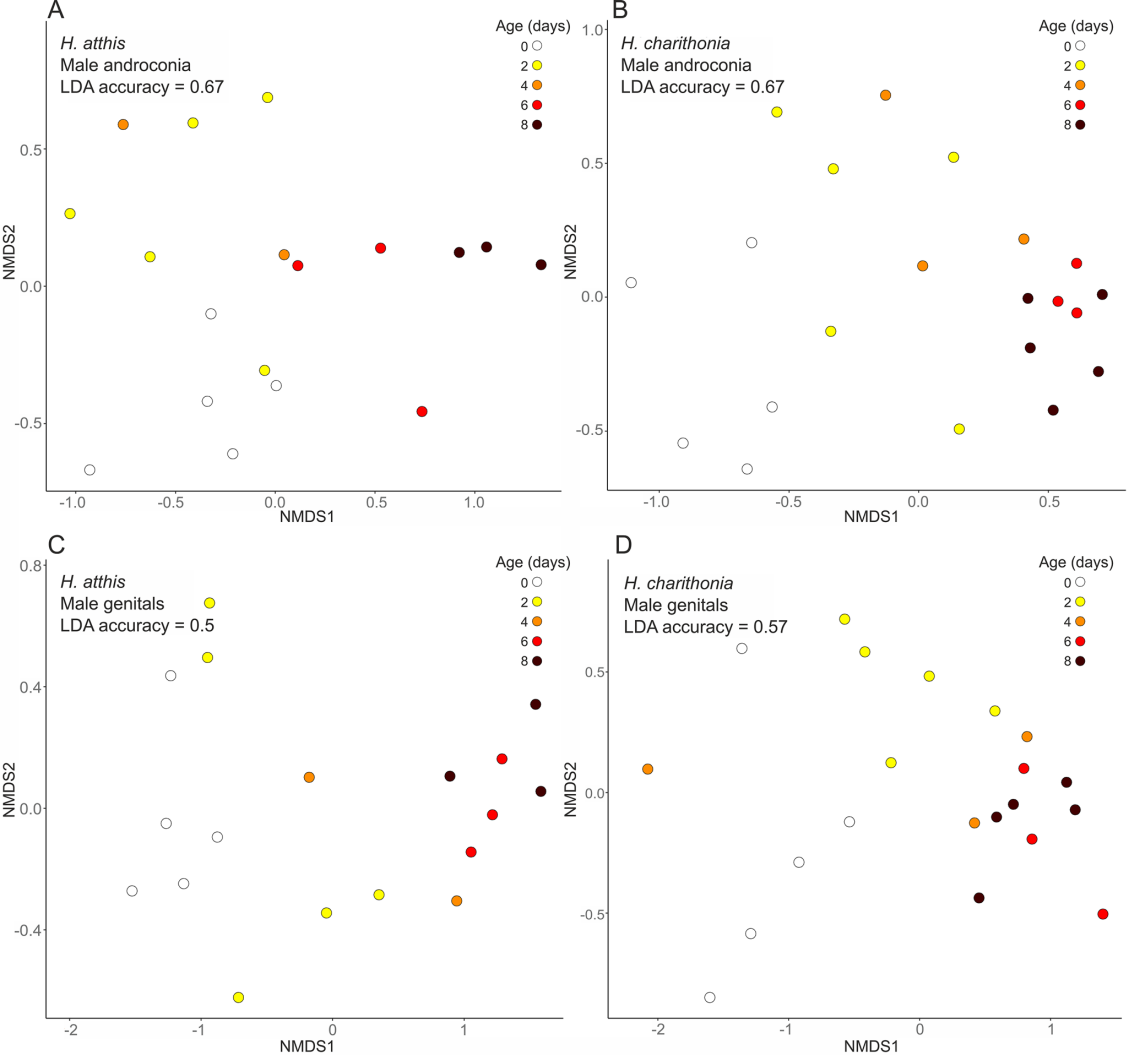
Change in chemical blends with age depicted using NMDS plots (k = 2, 1000 permutations) for *H. atthis* (**A**, **C**) and *H. charithonia* (**B**, **D**) male androconia and genitals. Colours represent samples of the same age. See Fig. S1 for NMDS plots for all six tissue types in each species

We utilized NMDS on the data from analysis of extracts of each species’ six tissue types as a dimensionality reduction technique to condense the data into a limited number of axes. We carried out an LDA on the first two NMDS axes (Fig. 2, Fig. S1). In general, chemical blend composition is a reasonable predictor of age, particularly when analysing extracts of the androconia of both species, but there are varying degrees of overlap in chemical content between different age classes for extracts of the same tissue (Fig. 2, Fig. S1). We found varying levels of predictability of the chemical composition based on age group, with the LDA models generally performing better on wing tissue extracts than on those of genital tissues (Table S1).

### Age Correlation and Tissue Specificity of Individual Compounds

**Fig. 3:**
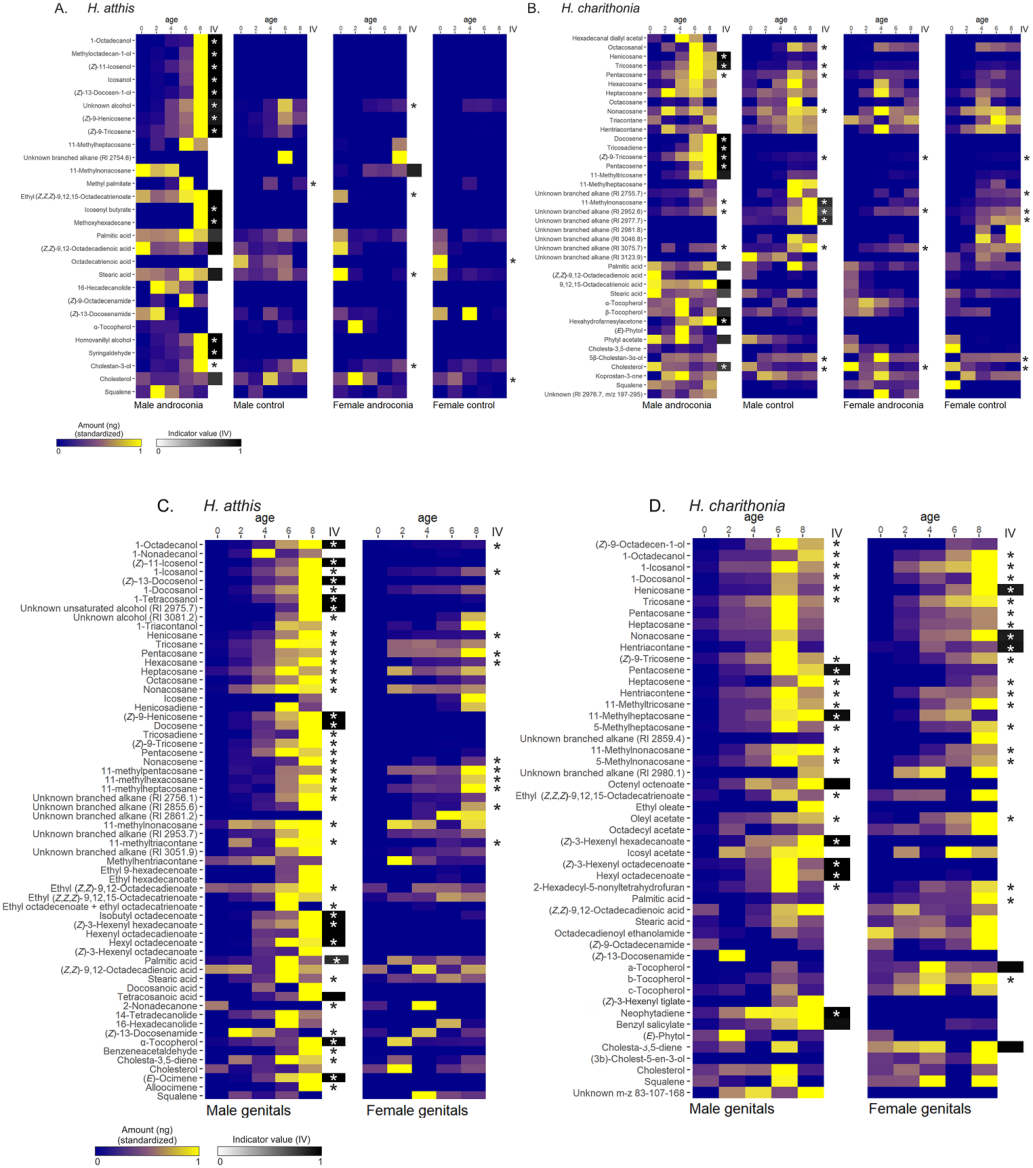
Changes in the relative amount with post-eclosion age for each compound detected in extracts of (**A**) H. atthis wing tissues, (**B**) H. charithonia wing tissues, (**C**) H. atthis genitals and (**D**) H. charithonia genitals. For each compound, the amount was standardized by dividing it by its maximum in that dataset. Compounds that show significant correlations with age are marked with an asterisk in each tissue type. The sixth column of each plot, labelled IV, shows the indicator value ($\sqrt{\text{specificity} \times \text{coverage}}$; Table 1) for each compound at 8 days post-eclosion within that tissue. Non-white tiles signify a high indicator value, making that compound an “indicator” within that tissue type. Compounds bearing both an asterisk and a high indicator value are potentially suitable candidates for the role of pheromones. Compounds are arranged by class, and then by retention index

Linear models that tested for the effect of age on the amount of each compound revealed that post-eclosion age significantly affected the amount of many individual compounds detected in the extracts (Fig. 3, see Table S3-4 for test statistics), which we refer to as age-dependent compounds. Age-dependent compounds were found in all examined tissues, and in both species and sexes. However, which compounds were affected by age varied greatly between the different tissues, sexes and species (Fig. 3).

We identified indicator compounds for several of the tissues featured in this study, reported in Table 1 alongside their tissue specificity (A) and sample coverage (B) values. Specificity indicates how specific the compound is to a tissue type, estimating the probability that the sample belongs to a certain tissue type based on the compound’s presence, with 1 meaning the compound is only found within that tissue type. Coverage indicates how widespread the compound is within a specific tissue type, with 1 meaning it is found within all samples of that tissue type. The indicator value ($IV=\sqrt{A\times B}$) (De Cáceres and Legendre 2009) for each detected compound, is represented in Fig. 3, also showing the overlap between indicator compounds and age-correlated compounds. Indicator compounds whose masses correlate positively with age so that they reach their full titre concurrently with sexual maturity may be particularly strong candidates for sex pheromone or antiaphrodisiac roles (Fig. 3). Most indicator compounds detected in *H. atthis* tissue extracts are specific to male tissues and their amounts are usually positively correlated with age within the same tissues. In *H. charithonia*, indicator compounds are more evenly distributed between the sexes, but fewer of them are correlated with age compared to *H. atthis* (Table 1).

**Table 1:** Significant indicator compounds for each tissue

A.	H. atthis wing tissues	Female “androconia”		Male androconia		Female control		Male control		
		A	B	A	B	A	B	A	B	Age effect
	1-Octadecanol	0.	0.	**1.**	**1.**	0.	0.	0.	0.	AM
	Methyloctadecan-1-ol	0.	0.	**1.**	**1.**	0.	0.	0.	0.	AM
	(*Z*)-11-Icosenol	0.	0.	**1.**	**1.**	0.	0.	0.	0.	AM
	Icosanol	0.	0.	**1.**	**1.**	0.	0.	0.	0.	AM
	(*Z*)-13-Docosen-1-ol	0.	0.	**1.**	**1.**	0.	0.	0.	0.	AM
	Unknown alcohol	0.09	0.75	**0.77**	**1.**	0.037	0.5	0.1	1.	AM-CM-CF
	(*Z*)-9-Henicosene	0.	0.	**0.82**	**1.**	0.	0.	0.18	1.	AM
	(*Z*)-9-Tricosene	0.	0.	**0.99**	**1.**	0.	0.	0.01	0.33	AM
	11-Methylnonacosane	**1.**	**0.75**	0.	0.	0.	0.	0.	0.	n.s.
	Ethyl (*Z,Z,Z*)-9,12,15-Octadecatrienoate	0.	0.	**1.**	**1.**	0.	0.	0.	0.	AF
	Icosenyl butyrate	0.	0.	**1.**	**1.**	0.	0.	0.	0.	AM-CF
	Methoxyhexadecane	0.	0.	**1.**	**1.**	0.	0.	0.	0.	AM
	Palmitic acid	0.14	1.	**0.62**	**1.**	0.087	1.	0.16	1.	n.s.
	(*Z,Z*)-9,12-Octadecadienoic acid	0.02	0.5	**0.98**	**1.**	0.	0.	0.	0.	n.s.
	Stearic acid	0.02	1.	**0.81**	**1.**	0.024	0.5	0.14	1.	AF
	Homovanillyl alcohol	0.	0.25	**1.**	**1.**	0.	0.	0.	0.	AM
	Syringaldehyde	0.	0.	**1.**	**1.**	0.	0.	0.	0.	AM
	Cholesterol	0.12	1.	**0.59**	**1.**	0.074	1.	0.21	1.	CF
B.	*H. charithonia* wing tissues									
	Henicosane	0.	0.	**0.97**	**1.**	0.	0.	0.03	0.4	AM
	Tricosane	0.03	0.67	**0.85**	**1.**	0.021	0.33	0.1	0.8	AM
	Docosene	0.	0.	**1.**	**1.**	0.	0.	0.	0.	AM
	Tricosadiene	0.	0.	**1.**	**1.**	0.	0.	0.	0.	AM
	(*Z*)-9-Tricosene	0.01	1.	**0.94**	**1.**	0.009	1.	0.04	1.	all
	Pentacosene	0.	0.	**0.98**	**1.**	0.	0.	0.02	0.2	AM
	11-Methyltricosane	0.01	0.67	**0.81**	**1.**	0.022	0.33	0.16	0.8	AM-CM
	11-Methylnonacosane	0.04	0.67	0.22	0.8	0.09	0.67	**0.65**	**1.**	AM-CM
	Unknown branched alkane (RI 2952.6)	0.13	1.	0.22	1.	0.205	1.	**0.44**	**1.**	AM-CM
	Unknown branched alkane (RI 2977.7)	0.	0.	0.	0.	0.365	1.	**0.63**	**1.**	CM
	Palmitic acid	0.07	0.33	**0.56**	**1.**	0.169	0.67	0.19	1.	n.s.
	9,12,15-Octadecatrienoic acid	0.	0.	**1.**	**1.**	0.	0.	0.	0.	n.s.
	Stearic acid	0.04	0.67	**0.49**	**1.**	0.174	0.67	0.3	0.8	n.s.
	β-Tocopherol	0.	0.	**0.67**	**1.**	0.	0.	0.33	0.6	n.s.
	Hexahydrofarnesylacetone	0.	0.	**1.**	**1.**	0.	0.	0.	0.2	AM
	Phytyl acetate	0.	0.	**0.82**	**0.8**	0.	0.	0.18	0.4	n.s.
	Cholesterol	0.2	1.	**0.54**	**1.**	0.065	0.67	0.2	0.8	all
C.	*H. atthis* genitals	Female genitals		Male genitals						
		A	B	A	B	Age effect				
	1-Octadecanol	0.04	1.	**0.96**	**1.**	GM-GF				
	(*Z*)-11-Icosenol	0.	0.	**1.**	**1.**	GM				
	(*Z*)-13-Docosenol	0.	0.	**1.**	**1.**	GM				
	1-Tetracosanol	0.01	0.25	**0.99**	**1.**	GM				
	Unknown unsaturated alcohol (RI 2975.7)	0.	0.	**1.**	**1.**	GM				
	(*Z*)-9-Henicosene	0.	0.	**1.**	**1.**	GM				
	Docosene	0.	0.	**1.**	**1.**	GM				
	Isobutyl octadecenoate	0.04	0.25	**0.96**	**1.**	GM				
	(*Z*)-3-Hexenyl hexadecanoate	0.	0.	**1.**	**1.**	GM				
	Hexenyl octadecadienoate	0.	0.	**1.**	**1.**	n.s.				
	Hexyl octadecenoate	0.	0.	**1.**	**1.**	GM				
	Tetracosanoic acid	0.	0.	**1.**	**1.**	GM				
	α-Tocopherol	0.05	0.75	**0.95**	**1.**	GM				
	(*E*)-Ocimene	0.	0.	**1.**	**1.**	GM				
D.	*H. charithonia* genitals									
	Henicosane	**0.81**	**1.**	0.19	1.	n.s.				
	Nonacosane	**0.8**	**1.**	0.2	1.	GF				
	Hentriacontane	**0.85**	**1.**	0.15	0.8	GF				
	Pentacosene	0.12	0.67	**0.88**	**1.**	GM				
	11-Methylheptacosane	0.07	0.33	**0.93**	**1.**	GM				
	(*Z*)-3-Hexenyl hexadecanoate	0.	0.	**1.**	**1.**	GM				
	(*Z*)-3-Hexenyl octadecenoate	0.	0.	**1.**	**1.**	GM				
	Hexyl octadecenoate	0.	0.	**1.**	**1.**	GM				
	Octenyl octenoate	0.	0.	**1.**	**1.**	n.s.				
	α-Tocopherol	**0.97**	**1.**	0.03	0.4	n.s.				
	Neophytadiene	0.	0.	**1.**	**1.**	n.s.				
	Benzyl salicylate	0.08	0.33	**0.92**	**1.**	GM				
	Cholesta-3,5-diene	**0.99**	**1.**	0.02	0.2	n.s.				

A = specificity, indicating the uniqueness of each compound to that specific tissue (1 = the compound is only found in that tissue). B = coverage, indicating the proportion of samples of that tissue the compound is found in (1 = the compound is found in all samples). Bold values indicate the tissue for which the compound is a suitable indicator (high values of A and B). The age effect columns report whether age was significantly correlated with that compound based on the linear models, and in what tissue; AM = male androconia, MC = male control, MG = male genitals, FA = female “androconia”, FC = female control, FG = female genitals, n.s. = no significant relationship with age in any tissue. Note that not all age-dependent compounds are indicator compounds and vice versa

- ***H. atthis *wing tissues. ***H. atthis* male androconia extracts contained more age-dependent compounds (13 in total) than all three control tissues (Fig. 3A). Of these, 12 are marked as indicator compounds within these tissues (Table 1A, Fig. 3A). Among these compounds, the most prevalent class is alcohols (both saturated and unsaturated), alongside Z9-alkenes and the aromatic compounds homovanillyl alcohol and syringaldehyde. Two fatty acids (palmitic and (*Z,Z*)-9,12-octadecadienoic acid) were also identified as indicator compounds in male androconia, but are not correlated with age. Conversely, very few age-dependent compounds were identified in the extracts of *H. atthis* wing control tissues. No indicator compounds were identified in either male or female wing control tissue extracts, and female “androconia” only have 11-methylnonacosane, which is unique to that tissue in *H. atthis* but has poor coverage (0.75) of samples and is not correlated with age.
- ***H. atthis *genitals. ***H. atthis* male genital extracts have 40 age-dependent compounds. However, only 13 of these are also indicators for this tissue (Table 1C, Fig. 3C). Age-dependent indicator compounds in *H. atthis* male genital extracts include the terpene (*E*)-β-ocimene, alkenes, esters and alcohols, as well as fatty acids. The presence of (*E*)-β-ocimene is especially noteworthy as this is a known antiaphrodisiac compound in *Heliconius melpomene*, wherein it is synthesized *de novo* (Darragh, Orteu, et al., 2019) and also appears in combination with a complex blend of higher molecular mass compounds (Schulz et al. 2008; Darragh, Orteu, et al., 2019). Any other volatile compounds that increase concurrently with the onset of sexual maturity might potentially have an analogous purpose to (*E*)-β-ocimene, or a synergistic effect with it (Schulz et al. 2008), collectively creating an antiaphrodisiac effect. Female genitals contain 12 compounds that significantly increase over time, two of which do so in a sex-specific manner, the long chain alkane nonacosane and (Z)-13-docosenamide. But none of these are indicator compounds, implying that most of the female products are also found in males.
- ***H. charithonia* wing tissues.** In *H. charithonia*, male androconia and male control have more age-dependent compounds than female “androconia” and female control (Fig. 3B). Similar to *H. atthis*, no indicator compounds were identified in either *H. charithonia* female wing tissue, implying no detected compounds were unique to females (Table 1B, Fig. 3B). Male control extracts have four indicator compounds (all large, branched alkanes that are likely cuticular components), all of which are age-dependent. Male androconia extracts have several indicator compounds, including straight-chain and branched alkanes, alkenes and the plant-derived hexahydrofarnesylacetone (Schulz et al. 2011). Three of these compounds (palmitic acid, stearic acid and cholesterol) are not age-dependent and have poor specificity, as they are detected in the extracts of the other three tissues as well. Most of the other male androconia indicator compounds also increase in correlation with age, and six of these showed this correlation exclusively in male androconia (Fig. 3B).
- ***H. charithonia *genitals.** While more age-affected compounds were identified in *H. charithonia* male androconia and control extracts than in the equivalent female tissue extracts, male and female genital extracts have comparable numbers of compounds whose amounts increase over time (22 in male genital extracts and 20 in female genital extracts), and most of these age-dependent compounds are common between the sexes. Male and female *H. charithonia* genital extracts also have comparable, though much lower, numbers of indicator compounds (Table S1D), and among these are most age-dependent compounds that are not shared between sexes: for males these consist of esters, pentacosene, 11-methylheptacosane and the aromatic benzyl salicylate, which may thus be suitable candidates for the role of anti-aphrodisiacs; for females, they consist mainly of long chain alkanes, typically components of the cuticle (Blomquist and Ginzel 2021). Female genital indicator compounds also include some non-age correlated sterols, which are also likely part of the cuticle (Clayton 1964) or possibly the internal genital tissues.

Most of the age-correlated compounds across species, sexes and tissue types increase in total mass over time, with few compounds showing a significant negative relationship between total mass and age. The negatively affected compounds were mostly (but not exclusively) fatty acids (Table S3-4). Palmitic, oleic and stearic acids are the primary precursors to most fatty acid (FA) derivatives in the dataset. Oleic acid was never detected. The amount of palmitic acid increased significantly with age in *H. atthis* male genital extracts, whereas that of stearic acid increased significantly with age in the genital extracts of *H. atthis* males and *H. charithonia* females, while it decreased significantly with age in *H. atthis* female “androconia” extracts. In extracts of all other tissue types, we found no correlation between age and FA precursor amount.

## Discussion

Androconial and genital chemical blend production is strongly age dependent in *Heliconius* regardless of the mating strategy. Post-eclosion age affects both the amount and the number of components detected in each tissue type. Shifts in the chemical profile of tissues begin before sexual maturity, with many compounds becoming detectable between day 2–4 (Figs. 1 and 3). While not all these compounds will have a behavioural effect, these results still imply that the organism requires time to biosynthesize the pheromone components needed to elicit a behavioural response, as opposed to the components already being present within the relevant tissues upon eclosion. This is not unusual in butterflies, where male chemical blends are usually incomplete immediately after eclosion and reach the correct titre and composition as the adult ages and feeds (Nieberding et al. 2008, 2012; Larsdotter-Mellström et al. 2012; Darragh et al. 2017). In most cases, we observed day 8 compounds appear by day 2 post-emergence. Regardless of the tissue or the sex, some compounds then begin increasing in amount.

As the observed increase in compound amount is not restricted to pheromone-emitting structures, one cannot assume that all compounds whose amount increases concurrently with the onset of sexual maturity have an active function in behavioural signalling. GC-MS of tissue extracts captures any soluble, volatile chemicals regardless of their role. Therefore, it is likely that some of these age-dependent compounds found in negative controls, such as the many branched alkanes seen in *H. charithonia* (Fig. 3B, Table 1B), are cuticular components not involved in sexual signalling, as changes in cuticle composition with age have been commonly demonstrated in other insects (Singer 1998; Cuvillier-Hot et al. 2001; Panek et al. 2001; Everaerts et al. 2010; Kuo et al. 2012; Heuskin et al. 2014). Due to the lack of behavioural testing, even volatile compounds that are normally not part of the cuticle’s composition cannot be assumed to have an active role as pheromones. Furthermore, fatty acid (FA) derivatives that make up *Heliconius* pheromone blends and cuticular hydrocarbons are produced via the same metabolic pathway (Liénard et al. 2014; Chung and Carroll 2015; Mann et al. 2017), meaning the pathway’s upregulation for the purpose of pheromone production as males approach sexual maturity may simultaneously lead to higher levels of cuticular hydrocarbons. This does not mean that age-dependent cuticular compounds have no function. Cuticle composition can indicate the sex and age of individuals, which in some cases are taken into account in mate choice (Nieberding et al. 2012; Heuskin et al. 2014), and behavioural effects of cuticular hydrocarbons are common (Howard and Blomquist 1982; Singer 1998; Panek et al. 2001; Lorenzi et al. 2004; Kuo et al. 2012; Chung and Carroll 2015).

Therefore, for a compound to be considered a good candidate as a male sex pheromone component, it would need not only to show a correlation between amount and age, but also to be found at significantly different levels in the genital or androconial extracts of mature males compared to females and wing control samples.

As such, the male androconia and male genitals columns in Table 1 report what might be putative pheromone components or matrix components that enhance the effects of the behaviourally-active compounds (Schulz et al. 2008). However, some of these compounds, like cholesterol and the tocopherol isomers, have rather high molecular mass and so are very unlikely to have a function as signalling volatiles, in addition to having known functions in other aspects of insect physiology, such as tocopherol (vitamin-E) serving as an antioxidant (Felton and Summers 1995), and cholesterol being involved in several metabolic pathways as well as being a cuticle component (Clayton 1964; Gimpl et al. 1997). It is important to clarify that even in the case of volatile compounds, this method to identify potential pheromone candidates cannot replace behavioural/electroantennographic assays as it does not offer any confirmation for the role of every compound in chemical signalling. However, we propose that when such data is still lacking, insight into which compounds vary concurrently with sexual maturity, combined with data on which compounds are exclusive to pheromone-producing structures, may offer a preliminary selection of candidate pheromone components to base further analyses on.

Nonetheless, based on past research on *Heliconius* chemical ecology, several of the compounds shown in Table 1 may potentially have behavioural activity or functions directly related to pheromone biosynthesis. Saturated and unsaturated alcohols (e.g. 1-octadecanol, (*Z*)-11-icosenol and (*Z*)*-*13-docosenol), alkanes (e.g. henicosane and tricosane) and alkenes ((*Z*)-9-henicosene and (*Z*)*-*9-tricosene) are frequently reported in *Heliconius* androconial extracts (Mann et al. 2017; Cama et al. 2022), and while this has never been validated via functional testing, their repeated detection within pheromone-producing tissues, where they occur in higher amount than in control tissues, may hint at a behavioural purpose within the genus. Alcohols are frequently involved in chemical signalling in Lepidoptera (Francke and Schulz 2010); for example, 1-octadecanol has a behavioural effect in *Heliothis virescens* (Hillier and Vickers 2004). It is also differentially produced between *Heliconius melpomene* and *Heliconius cydno*, which are closely-related and overlapping in their distributions, where it functions as the precursor to octadecanal, a compound that elicits strong behavioural responses and is heavily involved in chemical signalling during courtship (Byers et al. 2020). (*Z*)*-*11-Icosenol, while never tested for pheromonal activity within *Heliconius*, is a known pheromone in honeybees (Pickett et al. 1982). Δ9 and Δ11-desaturases, which are involved in the biosynthesis of compounds bearing (*Z*)-9- and (*Z*)-11- double bonds, are frequently active in Lepidopteran pheromone producing tissues (Tillman et al. 1999; Jurenka 2004; Francke and Schulz 2010; Yew and Chung 2015), meaning compounds bearing such double bonds may have active functions in chemical signalling. (*Z*)-9-Henicosene and (*Z*)*-*9-tricosene, and their saturated equivalents henicosane and tricosane, are differentially present in the *Heliconius elevatus*-*Heliconius pardalinus* sympatric species pair, where they are thought to contribute to species discrimination in mate choice (Rosser et al. 2019; Cama et al. 2022). Some of the age-dependent indicator compounds of male androconia are likely plant-derived metabolites; the phytol-derived hexahydrofarnesylacetone found in *H. charithonia* male androconia is a pheromone in *Pieris brassicae* butterflies (Schulz et al. 2011), and the aromatic compounds syringaldehyde and homovanillyl alcohol found in *H. atthis* male androconia have a known behavioural effect in beetles (Meyer and Norris 1967; Beggs et al. 2007). All of these metabolites frequently appear in *Heliconius* androconial extracts (Cama et al. 2022).

Little is known about the behavioural activity of most male genital compounds in *Heliconius*, but the terpene (*E*)-β-ocimene is a well-known antiaphrodisiac in *H. melpomene* (Schulz et al. 2008) and may therefore have a similar effect in *H. atthis.* No such compound was identified in *Heliconis charithonia* genital extracts, but both species’ genitals carry a variety of complex fatty acid esters such as (*Z*)*-*3-hexenyl hexadecanoate and hexyl octadecenoate; the presence of these esters is common in *Heliconius* genitals and they are speculated to modulate antiaphrodisiac function (Schulz et al. 2008; Estrada et al. 2011), a purpose which has been demonstrated in *H. melpomene* (Schulz et al. 2008), though it remains unverified in most species.

There is an overlap in compounds that increase with age in female *B. anynana* and in genital tissues of female *H. charithonia* and *H. atthis*; all of them display higher relative amounts of alkanes in later days post-eclosion compared to males (Heuskin et al. 2014). Changes in cuticular hydrocarbon profiles (including cuticular alkanes) with age are a well-documented phenomenon in insects, particularly in Diptera and Hymenoptera (Singer 1998; Lorenzi et al. 2004; Kuo et al. 2012), so it is unsurprising to find the same effect in Lepidoptera as well. Nonetheless, age-dependent cuticular hydrocarbons often have social implications for insects (Morel et al. 1988; Panek et al. 2001; Lorenzi et al. 2004; Kuo et al. 2012) and could potentially still have a role in mate choice, for example as seen in *Drosophila* (Kuo et al. 2012). It is possible that the age-dependent compounds detected in extracts of female tissues may have a role in short-range signalling, or even as contact pheromones (Würf et al. 2020), a still unexplored topic as *Heliconius* female pheromones have received little attention. However, a dilemma arises when we assume these changes have an active function in addition to being the result of an aging cuticle: female *Heliconius* reach sexual maturity soon after emergence, and immediately in the case of pupal maters like *H. charithonia* (Beltrán et al. 2007; Estrada et al. 2010), so why would their chemical signatures follow the same age-dependent pattern as that of males? If any of the female *Heliconius* compounds are involved in contact signalling, it may be deleterious to produce pheromones soon after emergence, potentially due to higher mortality in the early days and a greater investment being needed in survival rather than mating. Unfortunately, not enough is known about female *Heliconius* chemical signatures outside the context of pupal mating for us to be able to understand the reasons for this age effect.

Sample extracts of both species and sexes, and of all tissue types, contain compounds on day-0 post-eclosion, probably derived from stored larval nutrients (Larsdotter-Mellström et al. 2012). These day-0 compounds are not sex specific in either species with the exception of a few alkanes in *H. atthis* androconia. In fact, in both species the two sexes’ androconial chemical signatures post-eclosion are extremely similar, becoming more differentiated as age increases. Among the day-0 compounds, regardless of sample type, we find non-tissue specific fatty acids, alkanes, and occasionally very low amounts of tissue-specific compounds that are still detected in day-8 sample extracts, particularly in *H. charithonia* male androconia and genitals. These compounds are unlikely to have a behavioural function linked to pupal mating; none of them are *H. charithonia-*exclusive, and none of them are found among pupal secretions of either *H. charithonia* sex (Estrada et al. 2010). They may be related to energy storage and nutrition, as generally seen with sterols and polyunsaturated fatty acids in other insect species (Fraenkel and Blewett 1945; Moadeli et al. 2020).

The fatty acid metabolic pathway that allows many Lepidoptera (Bjostad and Roelofs 1981; Roelofs and Bjostad 1984; Liénard et al. 2014; Mann et al. 2017) to produce most of the compound classes that make up their pheromones is based on three main fatty acid (FA) precursors: the saturated palmitic acid (hexadecanoic acid, C16:0) and its elongation product stearic acid (octadecanoic acid, C18:0) and the unsaturated oleic acid ((*Z*)-9-octadecenoic acid, C18:1), obtained via Δ9 desaturation of stearic acid (Liénard et al. 2014; Mann et al. 2017). Using two species that produce both saturated and unsaturated FA derivatives, we were able to test whether these three precursors accumulate in the pheromone secreting tissues to facilitate their transformation into species-specific combinations of compounds. Palmitic and stearic acid are detected in day-0 extracts of all tissues tested, of both species, and their amounts tend to stay constant or increase over time (Table S3-S4), only showing a pattern of depletion in control wing tissues, not involved in pheromone signalling. Oleic acid, the unsaturated equivalent of stearic acid and the precursor to many unsaturated compounds (Mann et al. 2017), is entirely undetected in the dataset at all ages, whereas its unsaturated derivatives are detected, and increase over time (Table S3-S4). We cannot rule out the hypothesis that pheromones may be at least in part produced within the androconial and genital tissues; the FA precursor, rather than accumulating all at once to then subsequently be depleted, may be constantly supplied to the pheromone-emitting tissues, reaching an equilibrium between the rate of accumulation and depletion, a pattern potentially more consistent with the observed results (see palmitic, stearic and (*Z,Z*)-9,12-octadecenoic acid in Fig. 3).

However, in contrast with findings in *Bicyclus anynana* (Liénard et al. 2014), we have not found conclusive evidence that the FA components of the chemical blends in *Heliconius* androconia and genitals are preceded by accumulation of FA precursors in these tissues, These compounds may instead be stored elsewhere in the organism, most likely in the fat body, haemolymph or oenocytes (Tillman et al. 1999; Jurenka 2004; Howard and Blomquist 2005; Arrese and Soulages 2010), to be later transported to other tissues for further transformation, as seen in moths (Schal et al. 1998; Jurenka et al. 2003). Consistent with this, topical application of deuterium-labelled precursors (palmitic, oleic and stearic acid) to the androconia of *Heliconius hecale* (Cama 2022) did not lead to incorporation into its blend components, even though they were partially metabolized by the tissues and transformed into their ketone derivatives. Past work on *Drosophila melanogaster* has shown that the FA precursors to their cuticular hydrocarbon pheromones derive from multiple redundant sources (Wicker-Thomas et al. 2015), so the same could very well be true for *Heliconius*, and it may also explain the failure to detect oleic acid in our extracts. Furthermore, while fatty acids are common pheromone precursors in *Heliconius* (Mann et al. 2017), they are ubiquitous in animal tissues and function as energy storage in most organisms, so their presence in the organism soon after eclosion may be more indicative of that role rather than one in pheromone regulation.

Like several related *Heliconius* species, and almost uniquely across Lepidoptera, male *H. charithonia* engage in pupal mating (Gilbert 1976; Deinert et al. 1994; Beltrán et al. 2007), which makes courtship superfluous as males choose female pupae to guard rather than pursuing fully emerged mature females. For this reason, the timing of their androconial pheromones may not be as important as it is in a free-mating species like *H. atthis*, making it unnecessary for it to match their age of sexual maturity. However, regardless of mating strategy, the amount of male androconial and genital contents and the variety of synthesized chemical species increases with age, meaning it may be beneficial for pupal-mating species to also delay production of the full titre of pheromones.

Given these results, it is important to mention the extent of mating strategy differences between pupal and free-mating species. There is evidence pupal mating is not an obligate strategy in *Heliconius*; males will engage in this behaviour based on availability of female pupae, but are still capable of mating with a fully emerged female in the same way as a free-mating species would (Thurman et al. 2018). While free-mating species have a higher likelihood to mate more than once compared to pupal-mating species, mating events are still spaced out in time and most females mate only once regardless of mating strategy. An analysis of spermatophore counts demonstrated that only ~ 25% of wild-caught females from 17 free-mating species had remated (Walters et al. 2012) compared to 3% of females (not counting those carrying fully degraded, therefore uncountable, spermatophores) from 11 pupal-mating species. The sampling of pupal-mating species was also heavily biased towards *H. erato*, which showed a much more marked tendency for monogamy than the second most sampled species, *H. sara*, implying re-mating rates observed in *H. erato* might not be representative of other pupal-mating species (Walters et al. 2012). Therefore, less extreme differences in ecology and behaviour might be expected between *Heliconius* species with these different mating strategies compared to systems with obligate polyandry/monoandry. Since the two mating strategies are not always so behaviourally distinct, the evolution of differential timing for pheromone production may not necessarily be advantageous.

There are, however, sex-specific differences in compound production between the two species. The wing tissues of female *H. charithonia* accumulate more compound species over time compared to the relatively inert wing tissues of female *H. atthis* (Fig. 1, Table S2), and mature *H. charithonia* tissues contain a greater variety of compounds than those of *H. atthis* (Fig. 3). In fact, unlike *H. atthis*, all *H. charithonia* tissues, regardless of sex, showed a significant correlation between age and number of compounds detected (Table S2). This may imply a more active role of *H. charithonia* females (and potentially by extension, females of related species in the pupal-mating clade) in chemical signalling.

Lastly, it bears reminding that shifts in chemical composition with age can mediate many different types of social interactions in insects (Morel et al. 1988; Panek et al. 2001; Lorenzi et al. 2004). While the function of androconial and male genital emissions has been ascertained in *Heliconius* (Schulz et al. 2008; Darragh et al. 2017), age-dependent changes in chemical composition throughout all tissues may still have a role in intrasexual communication, a topic which remains completely unexplored in these butterflies.

Overall, aging revealed itself to be a complex topic in the context of *Heliconius* chemical ecology, and our work has highlighted how little is currently known about pheromone perception, which chemical components are behaviourally meaningful and important in female choice, and what factors, external or internal, might be controlling their relative amounts. With so many aspects left to investigate, there is no shortage of potential future directions for research into *Heliconius* chemical signalling.

### Electronic Supplementary Material

Below is the link to the electronic supplementary material.

Supplementary Material 1

Supplementary Material 2

Supplementary Material 3

## Declarations

### Data & Code Availability

Raw GC-MS files will be deposited and made available on Dryad. R code and datasets used to produce all figures and tables seen in the main text as well as in the Supplementary Information will be deposited on Zenodo, as well as Python scripts needed to operate the PyMassSpec package used for raw data processing.
