# Supplementary material for: Complexity of Chemical Emissions Increases Concurrently with Sexual Maturity in Heliconius Butterflies
Source: J Chem Ecol. 2024 Mar 13;50(5-6):197–213. doi: 10.1007/s10886-024-01484-z (PMC11233321; doi:10.1007/s10886-024-01484-z)
Supplement: Supplementary file 1 — Supplementary Material 1 [file 10886_2024_1484_MOESM1_ESM.pdf]

Complexity of Chemical Emissions Increases Concurrently with Sexual Maturity in *Heliconius* Butterflies (Supplementary Information 1)

Authors:

[1] Bruna Cama (bc716@york.ac.uk)

[2] Karl Heaton (karl.heaton@york.ac.uk)

[2] Jane Thomas-Oates (jane.thomas-oates@york.ac.uk)

[3] Stefan Schulz (stefan.schulz@tu-bs.de)

[1] Kanchon K. Dasmahapatra (kanchon.dasmahapatra@york.ac.uk)

[1] Department of Biology, University of York, Wentworth Way, Heslington, YO10 5DD, United Kingdom

[2] Department of Chemistry, University of York, Heslington, YO10 5DD, United Kingdom

[3] Institute of Organic Chemistry, Technische Universität Braunschweig, Hagenring 30, 38106 Braunschweig, Germany

Table S1. **LDA statistics on NMDS axes for both species and all tissue types. Loadings indicate each variable's contribution to the two LD functions, and "proportion of trace" refers to the proportion of data explained by each axis. The jackknifed accuracy of the LDA model is used to assess the fit of the model to the data and was obtained via leave-one-out (LOO) cross validation, ideal for smaller datasets.**

H. atthis	Loadings	LD1	LD2		Proportion of trace	Accuracy (jackknifed)
Male androconia	NMDS1	3.167	0.507	LD1	0.86	0.67
	NMDS2	2.822	-3.046	LD2	0.14	
Male control	NMDS1	-0.903	-1.045	LD1	0.84	0.61
	NMDS2	1.995	-0.969	LD2	0.16	
Male genitals	NMDS1	-2.336	-0.323	LD1	0.96	0.50
	NMDS2	-3.149	2.764	LD2	0.04	
Female "androconia"	NMDS1	0.375	1.777	LD1	0.63	0.85
	NMDS2	2.618	-0.671	LD2	0.37	
Female control	NMDS1	2.536	0.303	LD1	0.90	0.55
	NMDS2	-0.398	1.705	LD2	0.10	
Female genitals	NMDS1	2.358	-0.775	LD1	0.68	0.50
	NMDS2	-2.241	-3.016	LD2	0.32	
H. charithonia						
Male androconia	NMDS1	5.215	0.187	LD1	0.94	0.63
	NMDS2	-1.258	-3.539	LD2	0.06	
Male control	NMDS1	-7.441	-0.948	LD1	0.91	0.67
	NMDS2	-3.004	2.359	LD2	0.01	
Male genitals	NMDS1	2.630	-0.347	LD1	0.94	0.57
	NMDS2	-0.676	-2.258	LD2	0.06	
Female "androconia"	NMDS1	4.283	0.330	LD1	0.94	0.60
	NMDS2	-0.339	2.324	LD2	0.06	
Female control	NMDS1	10.264	-0.349	LD1	1.00	0.69
	NMDS2	-0.599	2.454	LD2	0.00	
Female genitals	NMDS1	2.705	0.154	LD1	0.88	0.50
	NMDS2	-0.613	-2.835	LD2	0.12	

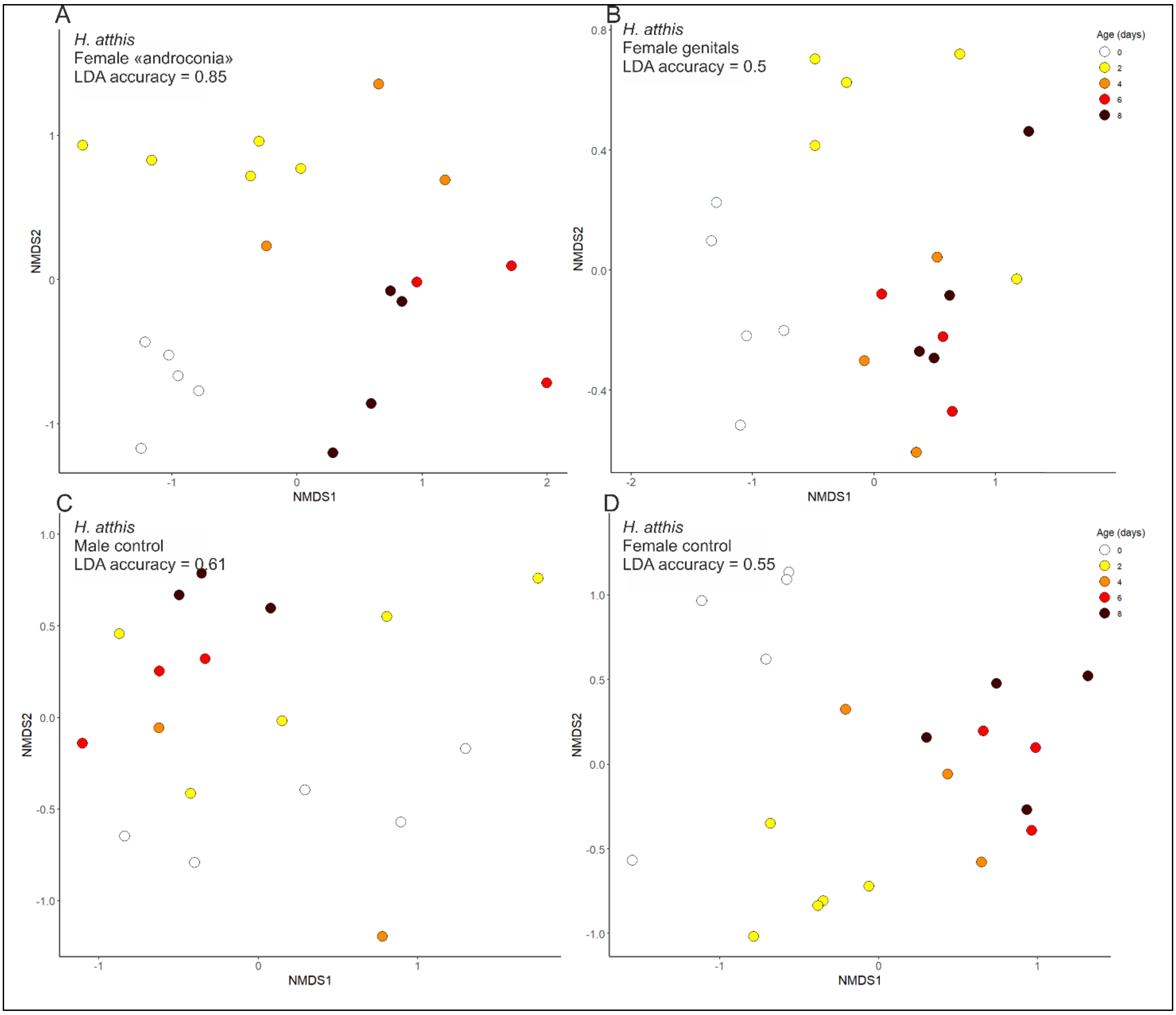

A
H. atthis
Female «androconia»
LDA accuracy = 0.85
NMDS1
NMDS2
B
H. atthis
Female genitals
LDA accuracy = 0.5
Age (days)
0
2
4
6
8
NMDS1
NMDS2
C
H. atthis
Male control
LDA accuracy = 0.61
NMDS1
NMDS2
D
H. atthis
Female control
LDA accuracy = 0.55
Age (days)
0
2
4
6
8
NMDS1
NMDS2

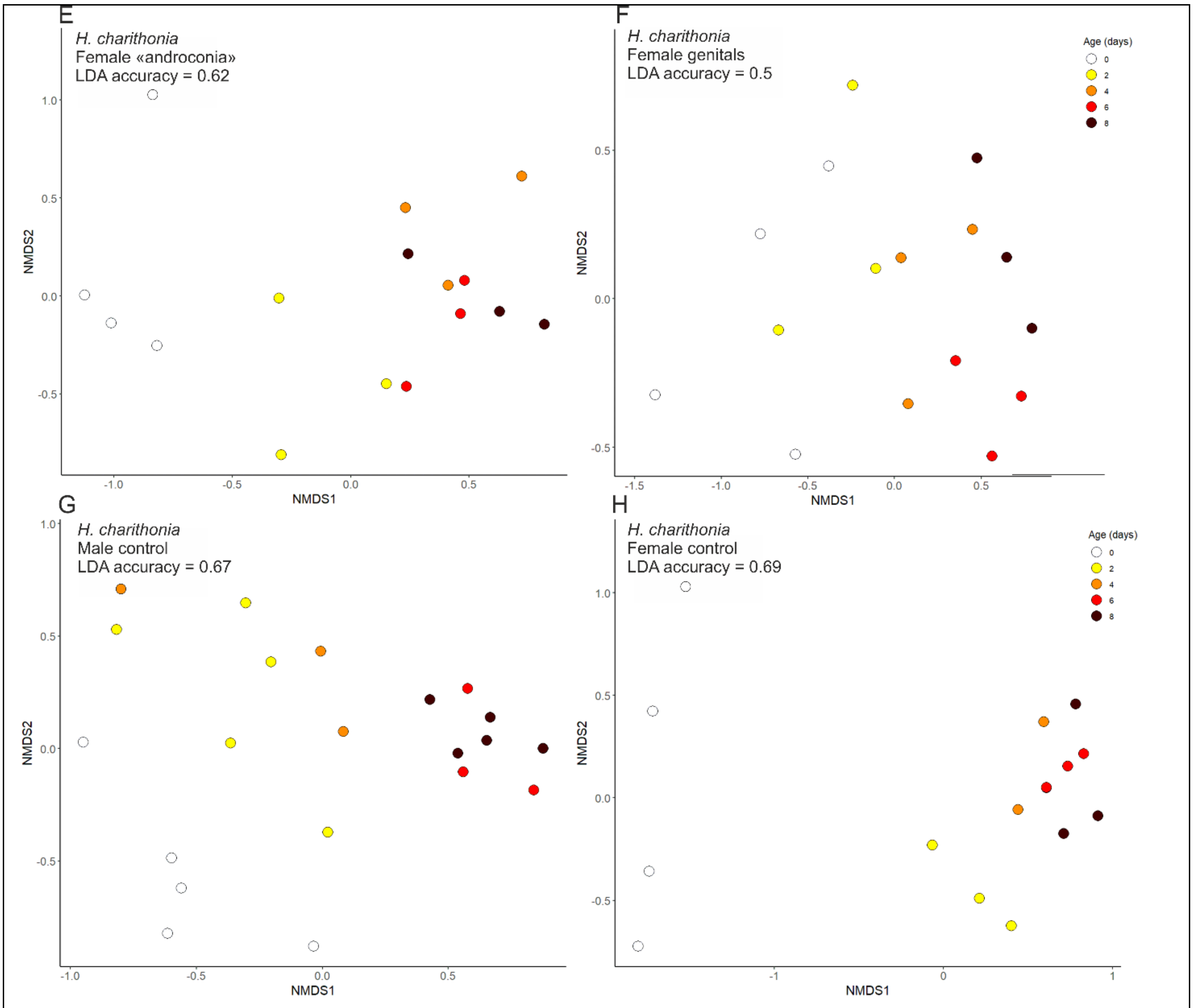

Figure S1. Change in chemical blends with age depicted using NMDS plots (k=2, 1000 permutations) for *H. atthis* (A-D) and *H. charithonia* (E-H) at all six tissue types (male androconia, male control, female "androconia", female control, male genitals, female genitals). Colours represent samples of the same age.

***Table S2.* Linear regression results for the effect of age on number of compounds detected and total amount of tissue contents in the six tissue types of *H. atthis* and *H. charithonia*. Values shown in bold show a significant correlation with age.**

H. atthis

	N compounds detected					Total amount of tissue contents (signal intensity)				
	R^2	t-value	F-statistic	df	p-value	R^2	t-value	F-statistic	df	p-value
**Male androconia**	**0.72**	**6.45**	**41.55**	**(1,16)**	**<0.001**	**0.55**	**4.43**	**19.64**	**(1,16)**	**<0.001**
**Male control**	**0.27**	**2.44**	**5.97**	**(1,16)**	**0.026**	0.01	0.45	0.20	(1,16)	0.658
**Male genitals**	**0.74**	**6.76**	**45.67**	**(1,16)**	**<0.001**	**0.82**	**8.57**	**73.53**	**(1,16)**	**<0.001**
Female "androconia"	0.07	1.16	1.35	(1,18)	0.261	0.17	-1.91	3.66	(1,18)	0.072
Female control	0.02	0.53	0.28	(1,18)	0.600	**0.40**	**-3.49**	**12.19**	**(1,18)**	**0.003**
**Female genitals**	**0.78**	**8.03**	**64.55**	**(1,18)**	**<0.001**	0.18	2.02	4.08	(1,18)	0.058

H. charithonia

	N compounds detected					Total amount of tissue contents (signal intensity)				
	R^2	t-value	F-statistic	df	p-value	R^2	t-value	F-statistic	df	p-value
**Male androconia**	**0.67**	**6.16**	**37.92**	**(1,19)**	**<0.001**	0.20	2.18	4.76	(1,19)	0.042
**Male control**	**0.56**	**4.92**	**24.17**	**(1,19)**	**<0.001**	0.03	0.73	0.53	(1,19)	0.475
**Male genitals**	**0.58**	**5.12**	**26.19**	**(1,19)**	**<0.001**	0.17	1.99	3.94	(1,19)	0.062
**Female androconia**	**0.41**	**3.12**	**9.76**	**(1,14)**	**0.007**	0.12	-1.37	1.88	(1,14)	0.191
**Female control**	**0.54**	**4.08**	**16.65**	**(1,14)**	**0.001**	0.22	-1.98	3.92	(1,14)	0.068
**Female genitals**	**0.72**	**6.00**	**35.98**	**(1,14)**	**<0.001**	**0.34**	**2.69**	**7.22**	**(1,14)**	**0.018**

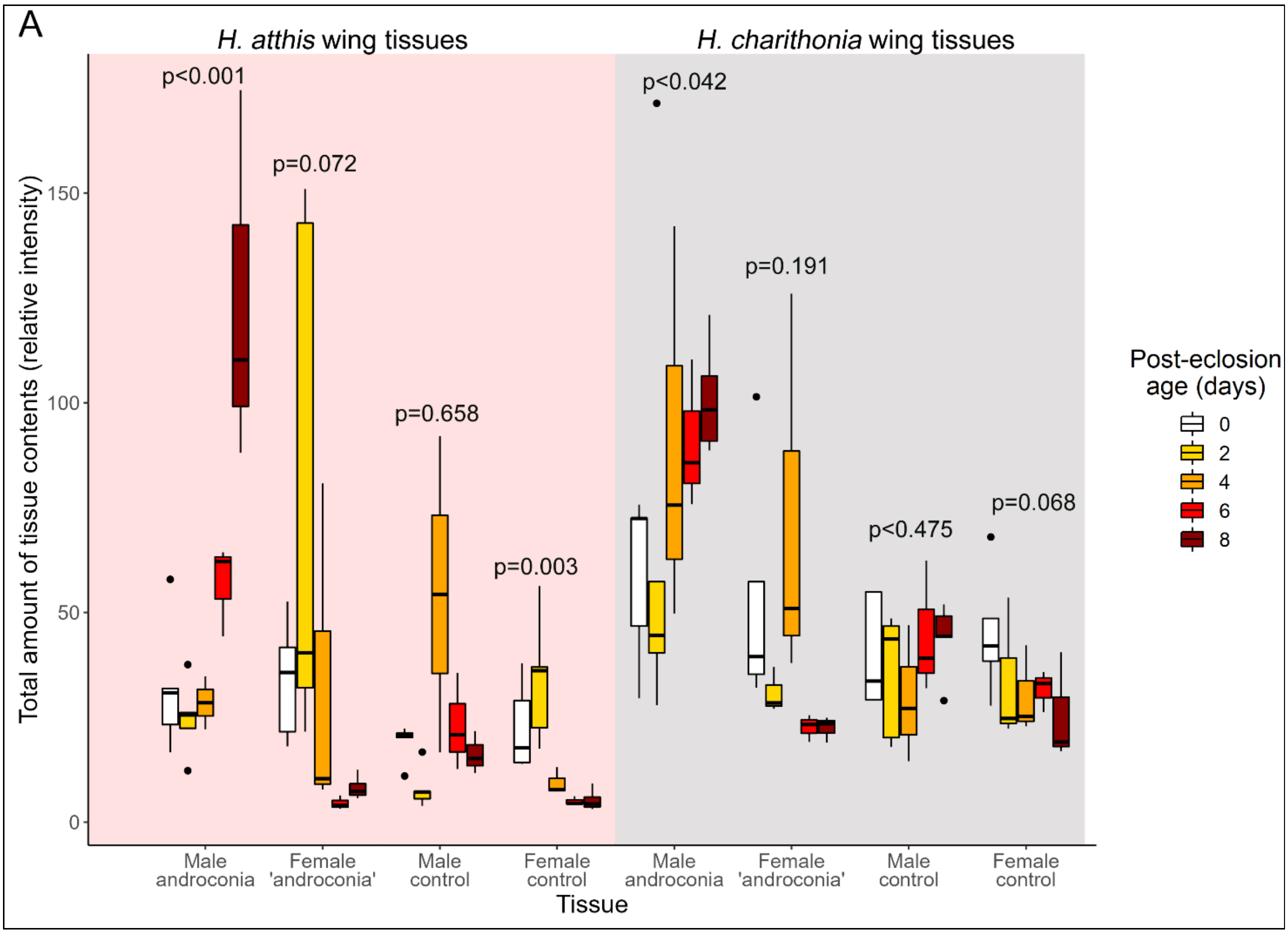
A
H. atthis wing tissues
H. charithonia wing tissues
p<0.001
p=0.072
p=0.658
p=0.003
p<0.042
p=0.191
p<0.475
p=0.068
Total amount of tissue contents (relative intensity)
0
50
100
150
Male androconia
Female 'androconia'
Male control
Female control
Male androconia
Female 'androconia'
Male control
Female control
Tissue
Post-eclosion age (days)
0
2
4
6
8

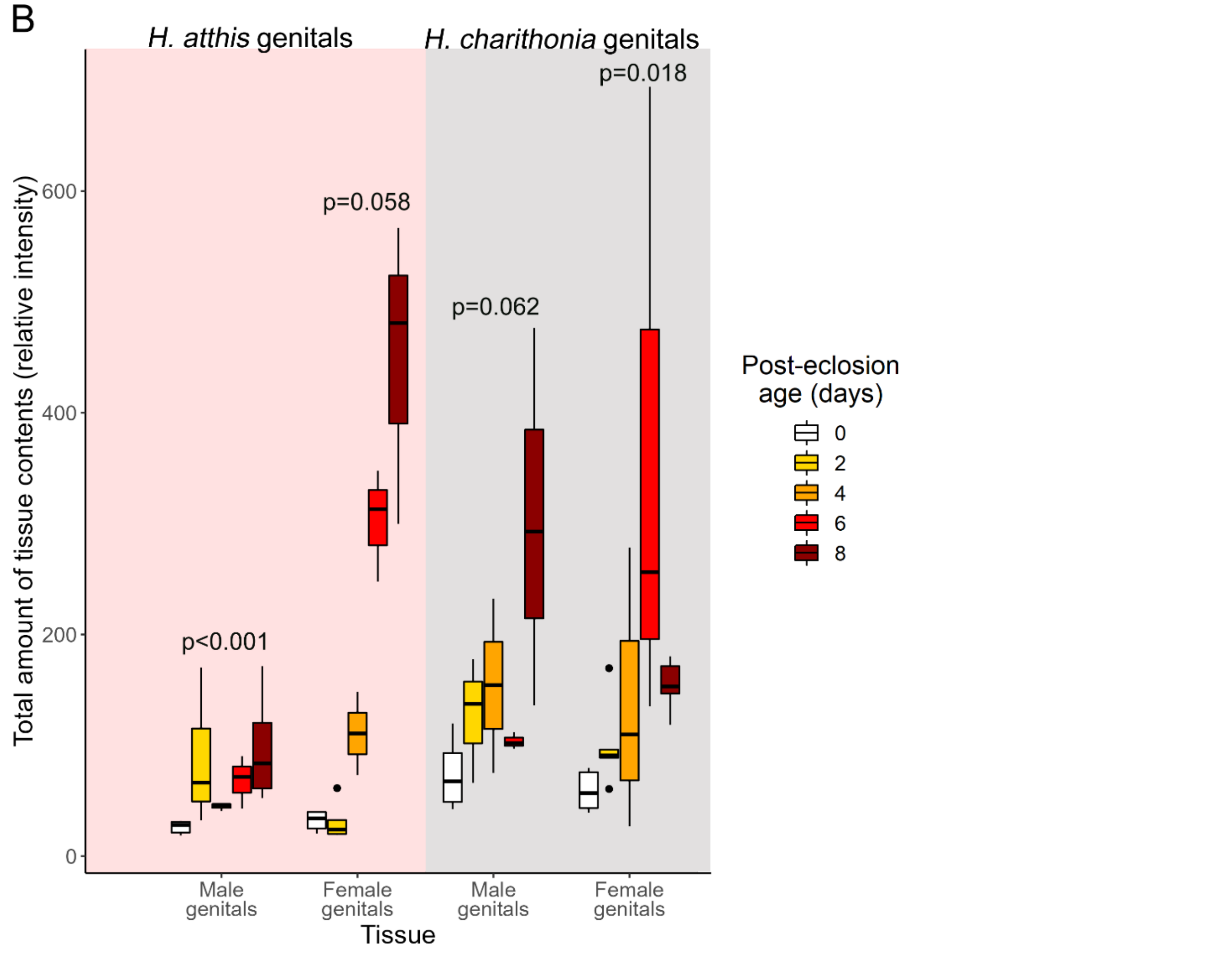

Figure S2. Effect of age on total amount of tissue contents detected in *H. atthis* and *H. charithonia* A) wing tissue extracts and B) genital extracts at all measured time points, also showing the data distribution for each time point in each tissue. Amount is expressed as intensity relative to the amount of internal standard (the latter is constant). Above each set are the linear regression *p*-values for the effect of age on number of compounds. See Table S2 for the complete linear regression results including all test statistics, as well as results for the effect of age on the total amount of all detected compounds.
